# Supplementary material for: Content-rich biological network constructed by mining PubMed abstracts
Source: BMC Bioinformatics. 2004 Oct 8;5:147. doi: 10.1186/1471-2105-5-147 (PMC528731; doi:10.1186/1471-2105-5-147)
Supplement: Additional File 2 — The original results of the above study (non-essential files are deleted to keep the file size under the limit set by BMC bioinformatics). [file 1471-2105-5-147-S2.bz2 › chilibotAdditionalFile2/dip50/721ID11799122E18058/html/ENVZ_OMPR.html]

 


 **ENVZ** and **OMPR** 
  
Found 178 abstracts in PubMed, retrieved 50.  
 

 What does Google say? 
 PDF only 
| .edu only 

---

**Interactive relationship** (e.g. stimulation, inhibition, etc)

**Neutral relationship**- The critical role of DNA in the equilibrium between  **OmpR**  and phosphorylated  **OmpR**  mediated by  **EnvZ**  in Escherichia coli.  Ref: 11158569 Proc Natl Acad Sci U S A, 2001
- Phosphorylation alters the interaction of the response regulator  **OmpR**  with its sensor kinase  **EnvZ** .  Ref: 11799122 J Biol Chem, 2002
- **EnvZ**  modulates the activity of  **OmpR**  by phosphorylation and dephosphorylation.  Ref: 9719640 J Mol Biol, 1998
- In the present work, we have used fluorescence anisotropy and phosphotransfer assays to examine  **OmpR**  interactions with  **EnvZ** .  Ref: 11799122 J Biol Chem, 2002
- **EnvZ**  can also dephosphorylate phospho  **OmpR**   **OmpR**  P to control the cellular level of  **OmpR**  P.  Ref: 11799122 J Biol Chem, 2002
- Interaction of  **EnvZ** , a sensory histidine kinase, with phosphorylated  **OmpR** , the cognate response regulator.  Ref: 12453215 Mol Microbiol, 2002
- **EnvZ**   **OmpR**  interaction and osmoregulation in Escherichia coli.  Ref: 11973328 J Biol Chem, 2002
- We also propose the existence of a specific signal, independent of  **EnvZ**  and AcP, leading to  **OmpR**  phosphorylation in response to pyruvate.  Ref: 10613880 J Bacteriol, 2000
- The osmosensor,  **EnvZ** , has dual enzymatic functions with  **OmpR**  kinase and  **OmpR**  P phosphatase.  Ref: 12453214 Mol Microbiol, 2002
- We demonstrated that  **OmpR**  P is able to interact with EnvZc with a similar affinity to  **OmpR**  and serves as an effective substrate for the  **EnvZ**  phosphatase.  Ref: 12453215 Mol Microbiol, 2002
- The classic sensor kinase  **EnvZ**  and acetyl phosphate collaborate to produce the optimum level of  **OmpR**  P needed for autoinduction.  Ref: 12068808 Mol Microbiol, 2002
- The expression of GST fusion proteins carrying the corresponding portions of  **EnvZ**  strongly interfered with the activation of porin gene expression by  **OmpR** .  Ref: 11325944 J Bacteriol, 2001
- The operon encodes two proteins,  **OmpR**  and  **EnvZ** , which share sequence identity with the  **OmpR**  and  **EnvZ**  proteins of a variety of other bacteria.  Ref: 10023081 Biochim Biophys Acta, 1999
- **OmpR**  P bound to EnvZc was readily released upon the addition of  **OmpR** , indicating that  **OmpR**  and  **OmpR**  P can compete for the binding to  **EnvZ** .  Ref: 12453214 Mol Microbiol, 2002
- At low osmolarity,  **OmpR**  P levels are low because of either low  **EnvZ**  kinase or high  **EnvZ**  phosphatase activities.  Ref: 11799122 J Biol Chem, 2002
- **EnvZ**  is a bifunctional enzyme that possesses both  **OmpR**  kinase and phosphorylated  **OmpR**   **OmpR**  P phosphatase activities and thus controls the cellular level of  **OmpR**  P.  Ref: 11158569 Proc Natl Acad Sci U S A, 2001
- Although both  **ompR**  and  **envZ**  genes are located on the ompB locus under the control of the ompB promoter and transcribed as a single polycistronic mRNA, the expression of  **envZ**  is known to be significantly less than  **ompR** .  Ref: 11973328 J Biol Chem, 2002
- The model predicts that when  **EnvZ**  is much less abundant than  **OmpR** , as is the case in E. coli, the steady state level of phosphorylated  **OmpR**   **OmpR**  P is insensitive to variations in the concentration of  **EnvZ** .  Ref: 12522261 Proc Natl Acad Sci U S A, 2003
- In an in vitro assay system, the addition of  **OmpR**  to the reaction mixture consisting of the cytoplasmic domain of  **EnvZ**  EnvZc and ATP produces a barely detectable amount of  **OmpR**  P because of the dual activities of  **EnvZ** .  Ref: 11158569 Proc Natl Acad Sci U S A, 2001
- We demonstrate that acetyl phosphate phosphorylates  **OmpR**  at aspartate 55, the same residue phosphorylated by the kinase  **EnvZ** .  Ref: 9719640 J Mol Biol, 1998
- We have generated a mutant form of the  **OmpR**  regulatory protein, OmpRD55E, that is active independent of the  **EnvZ**  kinase.  Ref: 9422609 J Bacteriol, 1998
- In Escherichia coli, the sensor kinase  **EnvZ**  detects a change in the osmotic environment and phosphorylates the response regulator  **OmpR** .  Ref: 10873450 J Mol Biol, 2000
- pta  **envZ**  double mutants had a residual level of porin transcription, implicating alternative means of  **OmpR**  phosphorylation under nutrient limitation.  Ref: 11700349 Microbiology, 2001
- **EnvZ**  is a sensory histidine kinase in Escherichia coli to regulate the phosphorylation of  **OmpR** , its cognate response regulator, required for the expression of genes for outer membrane porin proteins.  Ref: 12453215 Mol Microbiol, 2002
- Histidine kinase  **EnvZ** , a transmembrane osmotic sensor for Escherichia coli, is a bifunctional enzyme having  **OmpR**  its cognate response regulator kinase and phosphorylated  **OmpR**   **OmpR**  P phosphatase activities.  Ref: 12139613 Mol Microbiol, 2002
- **EnvZ** , the known cognate sensor kinase, and the porin genes known to be controlled by  **OmpR** , ompC and ompF, were not required for stationary phase ATR.  Ref: 10735868 J Bacteriol, 2000
- The histidine kinase phosphatase  **EnvZ**  helps Escherichia coli adapt to osmotic shock by controlling the phosphorylation state of the transcription factor  **OmpR** , which regulates the levels of the outer membrane porin proteins OmpF and OmpC.  Ref: 10973966 J Biol Chem, 2000
- The phosphorylation state of  **OmpR** , directed by the osmosensor  **EnvZ** , determines its ability to bind to the upstream regulatory regions of these genes, a total of 14 phospho  **OmpR**  binding sites.  Ref: 10096019 Genes Cells, 1998
- Here, for the first time, genetic evidence is provided that, in addition to  **EnvZ** , acetyl phosphate and an as yet unidentified sensor His kinase can serve as alternative in vivo phospho donors for  **OmpR** , even in the  **envZ**  background.  Ref: 10192921 Biosci Biotechnol Biochem, 1999
- **EnvZ** , a histidine kinase phosphatase in Escherichia coli, responds to the osmolarity changes in the medium by regulating the phosphorylation state of the transcription factor  **OmpR** , which controls the expression levels of outer membrane porin proteins OmpF and OmpC.  Ref: 11973328 J Biol Chem, 2002
- We propose that the apparent inhibitory effect of DNA on the  **EnvZ**  phosphatase function is caused by sequestrating  **OmpR**  P from the reaction as a result of  **OmpR**  P binding to DNA.  Ref: 11158569 Proc Natl Acad Sci U S A, 2001
- The autophosphorylation and  **OmpR**  kinase activities of the monomeric EnvZc AAB are similar to that of the wild type  **EnvZ** , while  **OmpR**  binding and phosphatase functions are reduced.  Ref: 10760160 Mol Microbiol, 2000

**Non-interactive relationship** (e.g. studied together, co-existance, homology, etc.)

- The SsrB regulon is modulated by the two component regulatory systems PhoP PhoQ and  **OmpR**   **EnvZ** , and is upregulated in the intracellular microenvironment.  Ref: 10844662 Mol Microbiol, 2000
- **EnvZ** , a histidine kinase, and its cognate response regulator  **OmpR**  of Escherichia coli are responsible for adaptation to external osmotic changes by regulating the levels of the outer membrane porin proteins, OmpF and OmpC.  Ref: 12453214 Mol Microbiol, 2002
- We found that  **ompR**  and  **envZ**  mutants were reduced or completely inhibited for the formation of Salmonella induced filaments Sifs.  Ref: 9529120 Infect Immun, 1998
- **EnvZ**  independent phosphotransfer signaling pathway of the  **OmpR**  mediated osmoregulatory expression of OmpC and OmpF in Escherichia coli.  Ref: 10192921 Biosci Biotechnol Biochem, 1999
- Neither trxB nor trxA affected gene regulation mediated by another dual component system tested,  **EnvZ**   **OmpR** .  Ref: 11114904 J Bacteriol, 2001
- Although hilC is regulated by all environmental conditions and regulatory factors that modulate hilA expression, hilC is not required for the regulation of hilA by any conditions or factors except  **EnvZ**   **OmpR** .  Ref: 11292791 J Bacteriol, 2001
- It was observed that during exponential growth in L broth medium there were approximately 3500 and 100 molecules per cell of  **OmpR**  and  **EnvZ** , respectively.  Ref: 11973328 J Biol Chem, 2002
- These findings support the hypothesis that osmotic signals regulate the level of the cellular concentration of  **OmpR**  P by modulating the ratio of kinase to phosphatase activity of the bifunctional enzymatic activities of  **EnvZ** .  Ref: 12453215 Mol Microbiol, 2002
- To test these predictions, we have perturbed the porin regulatory circuit in E. coli by varying the expression levels of  **EnvZ**  and  **OmpR** .  Ref: 12522261 Proc Natl Acad Sci U S A, 2003
- In Escherichia coli, porin gene expression is regulated, in part, by the two component regulatory system consisting of the two proteins  **EnvZ**  and  **OmpR** .  Ref: 9719640 J Mol Biol, 1998
- Although the order of the  **ompR**  and  **envZ**  genes of V. cholerae is similar to that of the ompB operon of E. coli, S. typhimurium and X. nematophilus, the Vibrio operon exhibits a number of novel features.  Ref: 10023081 Biochim Biophys Acta, 1999
- Mutants of the arcB, cyaA, lacZYA,  **ompR**   **envZ** , phnR, pstB, pstCA, pstS, pstSCAB phoU, recA, and torSTRCAD genes or operons were isolated as antibiotic resistant colonies after the introduction into bacteria carrying a Red expression plasmid of synthetic PCR generated DNA.  Ref: 10829079 Proc Natl Acad Sci U S A, 2000
- Under these conditions, the anaeroresponsive ArcB ArcA system, the osmoresponsive  **EnvZ**   **OmpR**  system and the response regulator UvrY showed the most drastic changes.  Ref: 12366850 Mol Microbiol, 2002
- The histidine kinase response regulator system  **EnvZ**   **OmpR**  of Escherichia coli regulates transcription of the genes ompF and ompC, encoding two porins of the outer membrane.  Ref: 11533042 J Biol Chem, 2001
- **OmpR**  and  **EnvZ**  comprise a two component system that regulates the porin genes ompF and ompC in response to changes in osmolarity.  Ref: 11799122 J Biol Chem, 2002
- Escherichia coli modulates its porin expression through a histidine kinase,  **EnvZ** , and its cognate response regulator,  **OmpR** .  Ref: 11158569 Proc Natl Acad Sci U S A, 2001
- The Escherichia coli  **EnvZ**   **OmpR**  regulatory system is a paradigm of intracellular signal transduction mediated by the well documented phosphotransfer mechanism, by which the expression of the major outer membrane proteins, OmpC and OmpF, is regulated in response to the medium osmolarity.  Ref: 10192921 Biosci Biotechnol Biochem, 1999
- A variety of genes hilC, hilD, fis, sirA barA, csrAB, phoB, fadD,  **envZ**   **ompR** , fliZ, hilE, ams, lon, pag, and hha have been identified that exert positive or negative effects on hilA expression, although the mechanisms by which these gene products function remain relatively unclear.  Ref: 12595445 Infect Immun, 2003
- In this study, isogenic transposon mutants of S. typhimurium with insertions in  **ompR** ,  **envZ** , ompF, ompC, ompD, osmZ, and tppB were compared with wild type SL1344 for trafficking in the human epithelial cell line HeLa.  Ref: 9529120 Infect Immun, 1998
- ii the two component system composed of  **OmpR**  regulator and  **EnvZ**  sensor regulates porin expression.  Ref: 10698756 Appl Environ Microbiol, 2000
- Formation of the stoichiometric complex of  **EnvZ** , a histidine kinase, with its response regulator,  **OmpR** .  Ref: 12453214 Mol Microbiol, 2002
- **EnvZ**  is autophosphorylated by intracellular ATP on a histidine residue, and it transfers the phosphoryl group to an aspartic acid residue of  **OmpR** .  Ref: 11799122 J Biol Chem, 2002
- The  **EnvZ**   **OmpR**  system in Escherichia coli, which regulates the expression of the porins OmpF and OmpC, is one of the simplest and best characterized examples of two component signaling.  Ref: 12522261 Proc Natl Acad Sci U S A, 2003
- Trafficking of porin deficient Salmonella typhimurium mutants inside HeLa cells  **ompR**  and  **envZ**  mutants are defective for the formation of Salmonella induced filaments.  Ref: 9529120 Infect Immun, 1998
- RESULTS Under standard aerobic growth conditions, it is well known that the osmoregulatory profile of the outer membrane porins OmpC and OmpF is mainly regulated by the  **EnvZ**   **OmpR**  phosphorelay system in response to medium osmolarity.  Ref: 10947842 Genes Cells, 2000
- Neither the transfer of the phosphoryl group from  **EnvZ**  approximately P to  **OmpR**  nor the  **EnvZ**  mediated  **OmpR**  approximately P dephosphorylation were affected by one of the tested solutes.  Ref: 11533042 J Biol Chem, 2001
- The influence of cyaA, rpoS, ackA and pta, as well as the two component  **envZ**   **ompR**  system, was studied under glucose and N limitation in chemostat cultures.  Ref: 11700349 Microbiology, 2001
- We now report a novel chromosomal bacteriolytic module of Escherichia coli called the entericidin locus, which is activated in stationary phase under high osmolarity conditions by sigmaS and simultaneously repressed by the osmoregulatory  **EnvZ**   **OmpR**  signal transduction pathway.  Ref: 9677290 J Mol Biol, 1998
- **EnvZ**  and  **OmpR**  are a transmembrane sensor and its cognate response regulator, respectively, regulating the transcription of porin genes in response to medium osmolarity in Escherichia coli.  Ref: 12754242 J Bacteriol, 2003
- The process is controlled by the histidine kinase  **EnvZ**  and the response regulator  **OmpR** .  Ref: 10518529 Proc Natl Acad Sci U S A, 1999
- In alpha proteobacteria, HPRK is located downstream of genes encoding a two component system of the  **EnvZ**   **OmpR**  family.  Ref: 12867744 J Mol Microbiol Biotechnol, 2003
- CONCLUSIONS Under anaerobic growth conditions, porin expression is tuned not only by the authentic osmo resposive  **EnvZ**  sensor, but also by the anaero responsive ArcB sensor, in an  **OmpR**  dependent manner.  Ref: 10947842 Genes Cells, 2000
- Among the four reactions occurring in the mixture, only the  **EnvZ**  phosphatase activity was inhibited severely by the specific DNA, in contrast to the previous report by Kenney and her associates that DNA stimulates  **OmpR**  phosphorylation by  **EnvZ**  Ames, S.  Ref: 11158569 Proc Natl Acad Sci U S A, 2001
- The operon flhDC is controlled by numerous regulators including H NS, CRP,  **EnvZ**   **OmpR** , QseBC and LrhA.  Ref: 12864862 Mol Microbiol, 2003
- The levels of  **OmpR**  and  **EnvZ**  in MC4100 cells grown in a high osmolarity medium nutrient broth with 20% sucrose were about the same as those grown in L broth.  Ref: 11973328 J Biol Chem, 2002
- Using these strains we have shown that, consistent with the predictions of our model, the transcription of ompC and ompF is indeed robust or insensitive to a wide range of expression levels of both  **EnvZ**  and  **OmpR** .  Ref: 12522261 Proc Natl Acad Sci U S A, 2003
- The  **ompR**   **envZ**  two component regulatory system has been shown to contribute to virulence in a number of enteric bacterial pathogens.  Ref: 9711851 FEMS Microbiol Lett, 1998
- The phosphoryl group of H6 HepK is transferred rapidly and efficiently to both H6DevR and H6DevRA but not to His tagged  **OmpR** , whose cognate sensor kinase is  **EnvZ** .  Ref: 12637541 J Biol Chem, 2003
- Partial sequence analysis of a large native plasmid approximately 120 kb, designated pA5H5, from a highly virulent Australian isolate revealed a putative two component regulatory system with inferred identity to the  **OmpR**  family of two component transcriptional regulatory proteins and  **EnvZ**  sensor kinases.  Ref: 12398213 Int J Med Microbiol, 2002
- Thus, we propose the existence of a new signal transduction pathway involved in the adherence process in addition to the  **EnvZ**   **OmpR**  two component system.  Ref: 10483736 FEMS Microbiol Lett, 1999
- Here we examined the levels of  **EnvZ**  and  **OmpR**  in the wild type strain MC4100 by quantitative Western blot analysis using anti  **OmpR**  and anti EnvZc cytoplasmic domain of  **EnvZ**  antisera.  Ref: 11973328 J Biol Chem, 2002
- The Escherichia coli  **OmpR**   **EnvZ**  two component regulatory system, which senses environmental osmolarity, also regulates biofilm formation.  Ref: 11717281 J Bacteriol, 2001
- Here, we demonstrate that the cytoplasmic kinase domain of  **EnvZ**  EnvZc and  **OmpR**  are able to form a 1 1 complex detected by native PAGE.  Ref: 12453214 Mol Microbiol, 2002
- Porin production was minimally dependent on  **EnvZ**  under N limitation due to  **OmpR**  phosphorylation by acetyl phosphate.  Ref: 11700349 Microbiology, 2001
- We conclude that  **OmpR**  and  **EnvZ**  appear to regulate Sif formation triggered by intracellular S. typhimurium.  Ref: 9529120 Infect Immun, 1998
- Outer membrane porin genes of Salmonella typhimurium, including ompC, ompF, and tppB, are regulated by the products of ompB, a two component regulatory locus encoding  **OmpR**  and  **EnvZ** .  Ref: 9529120 Infect Immun, 1998
- Furthermore, we establish that the antibiotic resistance phenotype is primarily due to the induction of the MarRAB regulatory system by the AOAs, while other regulatory pathways that also converge into micF modulation  **OmpR**   **EnvZ** , SoxRS, and Lrp remained unaltered.  Ref: 11353631 Antimicrob Agents Chemother, 2001
- Here, we show that FadD, FliZ, PhoB, and  **EnvZ**   **OmpR**  also require the URS to regulate hilA.  Ref: 11292791 J Bacteriol, 2001
- We speculate that  **EnvZ**   **OmpR**  regulates hilA by altering the expression and or activity of hilC, while all other regulatory conditions and mutations regulate hilA by modulating hilD posttranscriptionally.  Ref: 11292791 J Bacteriol, 2001
- In Escherichia coli, the two component regulatory system that controls the expression of outer membrane porins in response to environmental osmolarity consists of the sensor kinase  **EnvZ**  and the response regulator  **OmpR** .  Ref: 11812125 J Mol Biol, 2002
- Although it is clear that the  **EnvZ**  histidine His kinase is the major player in the phosphorylation of  **OmpR** , it has been assumed for some time that there may be an alternative phospho donor s that can phosphorylate  **OmpR**  under certain in vitro and in vivo conditions.  Ref: 10192921 Biosci Biotechnol Biochem, 1999
- The  **EnvZ**   **OmpR**  histidyl aspartyl phosphorelay HAP system in Escherichia coli regulates the expression of ompF and ompC, the major outer membrane porin genes, in response to environmental osmolarity changes.  Ref: 12767831 J Mol Biol, 2003
- Subdomain A, with a high helical content, contains the autophosphorylation site, H 243, and forms a stable dimer having the recognition site for  **OmpR** , the cognate response regulator of  **EnvZ** .  Ref: 9618480 Proc Natl Acad Sci U S A, 1998
- However, to date no accurate estimation for the amounts of  **EnvZ**  and  **OmpR**  in the cell has been carried out.  Ref: 11973328 J Biol Chem, 2002
- We have analyzed a mathematical model of the  **EnvZ**  mediated cycle of  **OmpR**  phosphorylation and dephosphorylation.  Ref: 12522261 Proc Natl Acad Sci U S A, 2003
- In this study, to address this long standing issue, extensive genetic studies were done with certain mutant alleles, including delta  **envZ** , delta ackA pta, and delta sixA, as well as delta  **ompR** .  Ref: 10192921 Biosci Biotechnol Biochem, 1999
- These results indicate that curli are morphological structures of major importance for inert surface colonization and biofilm formation and demonstrate that their synthesis is under the control of the  **EnvZ**   **OmpR**  two component regulatory system.  Ref: 9573197 J Bacteriol, 1998
- Here, we re examined the recent paper Mattison and Kenney, in which the authors reported that phosphorylated  **OmpR**   **OmpR**  P is unable to bind to  **EnvZ** , thus casting doubts on the role of the  **EnvZ**  phosphatase activity in vivo.  Ref: 12453215 Mol Microbiol, 2002
